# Statin Use May Be Associated With Reduced Active Tuberculosis Infection: A Meta-Analysis of Observational Studies

**DOI:** 10.3389/fmed.2020.00121

**Published:** 2020-04-24

**Authors:** Xiaofei Li, Lina Sheng, Lanqing Lou

**Affiliations:** Department of Infectious Diseases, Yiwu Central Hospital, Yiwu, China

**Keywords:** statin, tuberculosis, infection, diabetes, meta-analysis

## Abstract

**Background:** Tuberculosis remains one of the leading causes of mortality among the infectious diseases, while statins were suggested to confer anti-infective efficacy in experimental studies. We aimed to evaluate the association between statin use and tuberculosis infection in a meta-analysis.

**Method:** Relevant studies were obtained via systematically search of PubMed and Embase databases. A random or a fixed effect model was applied to pool the results according to the heterogeneity among the included studies. Subgroup analyses according to the gender and diabetic status of the participants were performed. We assessed the quality of evidence with the Grading of Recommendations Assessment, Development and Evaluation (GRADE) approach.

**Results:** Nine observational studies were included. Significant heterogeneity was detected among the studies (p for Cochrane's Q test <0.001, *I*^2^ = 93%). The GRADE approach showed generally low quality of evidence. Pooled results showed that statin use was associated with reduced active tuberculosis infection (risk ratio [RR]: 0.60, 95% confidence interval [CI]: 0.45 to 0.75, *p* < 0.001). Subgroup analyses showed that the negative association between statin use and active tuberculosis infection was consistent in men (RR: 0.63, *p* = 0.01) and women (RR: 0.58, *p* < 0.001), in participants with (RR: 0.63, *p* = 0.02) and without diabetes (RR: 0.50, *p* < 0.001), in retrospective cohort studies (RR: 0.56, *p* = 0.02), prospective cohort studies (RR: 0.76, *p* = 0.03), nested case-controls studies (RR: 0.57, *p* < 0.001), and case-control studies (RR: 0.60, *p* < 0.001), and in studies with statin used defined as any use within 1 year (RR: 0.59, *p* < 0.001) or during follow-up (RR: 0.61, *p* < 0.001). Significant publication bias was detected (p for Egger's regression test = 0.046). Subsequent “trim and fill” analyses retrieved an unpublished study to generate symmetrical funnel plots, and meta-analysis incorporating this study did not significantly affect the results (RR: 0.72, 95% CI: 0.68 to 0.76, *p* < 0.001).

**Conclusions:** Statin use may be associated with reduced active tuberculosis infection. Randomized controlled trials (RCTs) are needed to confirm the potential preventative role of statin use on tuberculosis infection.

## Introduction

Despite of great efforts in protective inoculation and treatment, tuberculosis remains one of the leading causes of mortality among the infectious diseases (1). According to the report of the World Health Organization (WHO), more than 10 million new cases of tuberculosis infection were diagnosed globally in 2017 (1). The conventional antituberculosis regimens include long-term use of multiple medications with inevitable drug-related adverse effects, which further leads to a poor adherence (2, 3). However, the long-term mortality remains high for patients who have received antituberculosis treatment (4). Moreover, in 5~25% cases, the tuberculosis infection may be drug resistant (5). Therefore, identification of protective strategies against tuberculosis infection remains important in current clinical practice.

Statins are a category of conventionally used cholesterol lowering medications. By targeted inhibition the synthesis of cholesterol, statins have been applied as the cornerstone medications for the primary and secondary prevention of coronary artery disease (6). Interestingly, accumulating evidence revealed many other potential pharmacological effects of statins besides their lipids-lowering efficacy, such as anti-inflammation, anti-oxidative stress, immune regulation, and possibly anti-infection (7). Evidence from experimental studies showed that statins could enhance the immune response of the host toward *Mycobacterium tuberculosis* (*M. tuberculosis*) infection (8). Moreover, statins may also synergistically increase the treatment efficacy of antituberculosis, such as rifampin (9). However, an early cohort study did not show a significant association between statin use and tuberculosis infection (10), while subsequent studies indicated that use of statins may be associated with reduced tuberculosis infection (11–18). The potential reasons for the inconsistencies of the above findings remain unknown. Therefore, we aimed to perform a meta-analysis to systematically evaluate the association between statin use and tuberculosis infection. Moreover, the influences of participant characteristics on the outcome, such as the gender and diabetic status, were also explored.

## Methods

The meta-analysis was performed in accordance with the MOOSE (Meta-analysis of Observational Studies in Epidemiology) (19) and Cochrane's Handbook (20) guidelines.

### Literature Search

Studies were identified via systematic search of electronic databases of PubMed and Embase via the following terms: (1) “statin” OR “3-hydroxy-3-methyl-glutarylCoA reductase inhibitor” OR “CS-514” OR “statin” OR “simvastatin” OR “atorvastatin” OR “fluvastatin” OR “lovastatin” OR “rosuvastatin” OR “pravastatin” OR “pitavastatin”; and (2) “tuberculosis” OR “tubercle bacillus” OR “mycobacterium tuberculosis” OR “TB” OR “mycobacteria” OR “antituberculosis.” The search was limited to human studies with no restriction of languages. The reference lists of related original and review articles were also analyzed using a manual approach. The final literature search was performed on September 15, 2019.

### Study Selection

The inclusion criteria for the studies were: (1) observational studies published in full-length articles; (2) included patients with and without statin use at baseline; (3) evaluated the association between statin use and active tuberculosis infection; and (4) reported the relative risk for the association after adjustment of potential confounding factors. Diagnosis of active tuberculosis infection was in accordance with the criteria adopted in each study. Reviews, editorials, preclinical studies, and studies irrelevant to the aim of current meta-analysis were excluded.

### Data Extracting and Quality Evaluation

Literature search, data extraction, and quality assessment of the included studies were performed according to the predefined inclusion criteria. Discrepancies were resolved by consensus. The extracted data included: (1) name of first author, publication year, and country where the study was performed; (2) study design characteristics; (3) ethnicity, characteristics, age, and gender of the participants; (4) definition of statin use; (5) follow-up durations for cohort studies; (6) validation of active tuberculosis infection and number of patients with tuberculosis infection; and (7) variables adjusted when presenting the results. The quality of each study was evaluated using the Newcastle-Ottawa Scale (21) which ranges from 1 to 9 stars and judges each study regarding three aspects: selection of the study groups; the comparability of the groups; and the ascertainment of the outcome of interest. Moreover, we used the Grading of Recommendations Assessment, Development and Evaluation (GRADE) approach to assess the quality of the body of evidence (22). The GRADE methodology (23) involves rating the initial quality of observational data as “low,” followed by upgrading based on three criteria (large effect size, dose-response gradient, and plausible confounding).

### Statistical Analyses

We used risk ratios (RRs) and their corresponding 95% confidence intervals (CIs) as the general measure for the association between statin use and infection of tuberculosis. Data of RRs and their corresponding stand errors (SEs) were calculated from 95% CIs or *p*-values, and were logarithmically transformed to stabilize variance and normalized the distribution (20). The Cochrane's Q test and I^2^ test were used to evaluate the heterogeneity among the include cohort studies (24). A significant heterogeneity was considered if I^2^ > 50%. A random-effect model was used to pool the results if significant heterogeneity was detected among the included studies; otherwise, a fixed-effect model was applied, in accordance with the Cochrane's Handbook for Systematic Review and Meta-analysis (20). Sensitivity analyses, by removing one individual study at a time, were performed to test the robustness of the results (25). Predefined subgroup analyses were performed to evaluate the influences of patient characteristics (gender, with or without diabetes), study design, and definitions of statin use on the outcome. The potential publication bias was assessed by funnel plots with the Egger's regression asymmetry test (26). If publication bias was detected, we used the “trim-and-fill” analyses to evaluate the potential influence of imputed unpublished studies with negative results on the outcome (20). This method incorporated the hypothesized unpublished studies to generate symmetrical forest plots. We used the RevMan (Version 5.1; Cochrane Collaboration, Oxford, UK) and STATA software for the meta-analysis and statistics.

## Results

### Literature Search

**Figure 1:**
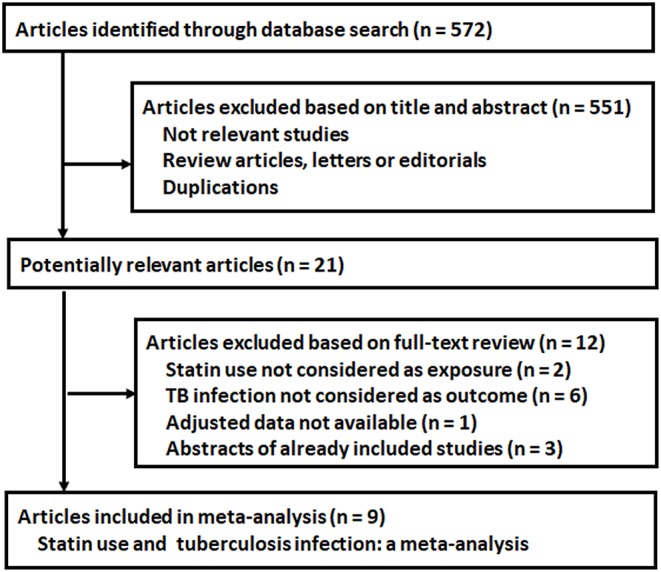
Flowchart of database search and study identification.

The process of database search was summarized in (Figure 1). Briefly, 572 articles were found via initial literature search of the PubMed and Embase databases, and 551 were excluded through screening of the titles and abstracts mainly because they were not relevant to the purpose of the meta-analysis. Subsequently, 21 potential relevant records underwent full-text review. Of these, 12 were further excluded because two of them did not treat statin use as the exposure, six did not report tuberculosis infection as the outcome, one study did not provide available outcome data, and the other three were abstracts of already included studies. Finally, nine observational studies were included (10–18).

### Study Characteristics and Quality Evaluation

The characteristics of the included studies were summarized in (Table 1). Overall, nine observational studies with a total of 2,133,735 participants were included. These studies were published between 2014 and 2019, and all of them included Asian people. Regarding the study design, five studies were retrospective cohort studies (10, 15–18), one was a prospective cohort study (11), two were nested case-control studies (12, 14), and another one was a retrospective case-control study (13). As for the characteristics of the included patients, four of them included diabetic patients (10, 11, 15, 18), while three studies provided data stratified by the diabetic status (12, 14, 17). The mean ages of the patients varied between 51 and 65 years, with the proportions of male ranging from 44 to 70%. Statin use was defined as any stain use within 1 year before the end of follow-up in six studies (10, 12–15, 17), and any stain use during follow-up in three studies (11, 16, 18). The follow-up durations varied from 1 to 12 years, and the outcome of active tuberculosis infection was validated via the International Classification for Diseases codes and records of antituberculosis prescription. A total of 23,782 cases of active tuberculosis infection were included. Potential confounding factors, such as age, gender, comorbidities, and using of other medications, were adjusted when presenting the outcome in all of the included studies. The NOS scores of the included studies ranged were six for two studies, and seven for seven studies, which were presented in detail in Table 2. Following the GRADE methodology, we graded the quality of evidence for the outcome “risk of active tuberculosis infection” to be low because risk of bias of inconsistency and indirectness may exist (Table 3).

**Table 1:** Characteristics of the included studies.

**Study**	**Design**	**Country**	**Patient characteristics**	**Sample size**	**Mean age**	**Male**	**Definition of statin use**	**Controls**	**Follow-up duration**	**TB infection validation**	**TB cases**	**Variables adjusted**	**NOS**
					years	%			years				
Kang (10)	RC	Korea	T2DM patients	840,899	56.3	59.1	Any statin use within 1y before the end of follow-up	No statin use within 1y before the end of follow-up	mean:1.9	ICD-10 and anti-TB medication prescription	4,052	Age, sex, history of malignancies, HIV/AIDS, other comorbidities, and antidiabetics	7
Lee (11)	PC	China	T2DM patients > 65 years	13,981	NA	46.1	Any statin use during follow-up	No statin use during follow-up	1~12	ICD-9 and prescription of anti-TB medication for > 28 days	286	Age, sex, AIDS, other co-morbidities and medications	7
Lai (12)	NCC	China	Adult population	817,898	60.3	68.8	Any statin use for > 90d within 1y before the end of follow-up	No statin use within 1y before the end of follow-up	9.8	ICD-9 and prescription of anti-TB medication for > 28 days	8,098	Age, sex, other risk factors for TB, and other medications	7
Su (14)	NCC	China	Adult population	305,142	NA	50.7	Any statin use for > 30d within 1y before the end of follow-up	No statin use within 1y before the end of follow-up	5.6	ICD-9 and prescription of anti-TB medication for > 28 days	1,264	Age, sex, urbanization level, other risk factors for TB, and other medications	7
Liao (13)	CC	China	Adult population	16,472	59.3	69.4	Any statin use within 1y before the end of follow-up	No statin use within 1y before the end of follow-up	NA	ICD-9 and anti-TB medication prescription	8,236	Age, sex, other risk factors for TB, and medications	7
Yeh (16)	RC	China	ACOS patients	11,256	64.1	55.3	Any statin use during follow-up	No statin use during follow-up	7.1	ICD-9 and anti-TB medication prescription	551	Age, sex, comorbidities and use of other medications	6
Lin (15)	RC	China	T2DM patients	49,028	51.2	50.6	Any statin use within 1y before the end of follow-up	No statin use within 1y before the end of follow-up	1~11	ICD-9 and prescription of anti-TB medication for > 90 days	917	Age, sex, DM duration, comorbidities and use of other medications	7
Kim (17)	RC	Korea	Adult population	56,036	52.5	49	Any statin use for > 7d within 1y before the end of follow-up	No statin use	11	ICD-10 and anti-TB medication prescription	265	Age, sex, comorbidities and use of other medications	7
Pan (18)	RC	China	T2DM patients	23,023	54.5	44.1	Any statin use during follow-up	No statin use	5.6	ICD-9 and prescription of anti-TB medication for > 28 days	113	Age, sex, severity of DM, comorbidities and use of other medications	6

*TB, tuberculosis; NCC, nested case-control; CC, case-control; PR, prospective cohort; RC, retrospective cohort; T2DM, type 2 diabetes mellitus; DM, diabetes mellitus; NA, not available; ICD, International Classification for Diseases; DM, diabetes mellitus; HIV, human immunodeficiency virus; AIDS, acquired immune deficiency syndrome; ACOS, asthma–chronic pulmonary disease overlap syndrome*.

**Table 2:** Details of quality evaluation via the Newcastle-Ottawa Scores.

**Cohort studies**	**Representativeness of the exposed cohort**	**Selection of the non-exposed cohort**	**Ascertainment of exposure**	**Outcome of interest not present at baseline**	**Adjustment of age and gender**	**Adjustment of other confounding factors**	**Assessment of outcome**	**Follow-Up long enough**	**Adequacy of follow-up of cohorts**	**Total**
Kang (10)	No	Yes	Yes	Yes	Yes	Yes	Yes	No	Yes	7
Lee (11)	No	Yes	No	Yes	Yes	Yes	Yes	Yes	Yes	7
Yeh (16)	No	Yes	No	Yes	Yes	No	Yes	Yes	Yes	6
Lin (15)	No	No	Yes	Yes	Yes	Yes	Yes	Yes	Yes	7
Kim (17)	Yes	No	No	Yes	Yes	Yes	Yes	Yes	Yes	7
Pan (18)	No	Yes	No	Yes	Yes	No	Yes	Yes	Yes	6
Case-control studies	Adequate definition of case	Representativeness of the cases	Selection of controls	Definition of controls	Adjustment of age and gender	Adjustment of other confounding factors	Ascertainment of exposure	Same method for ascertainment of case and control	Non-response rate	Total
Lai (12)	Yes	No	Yes	Yes	Yes	Yes	Yes	Yes	No	7
Su (14)	No	No	Yes	Yes	Yes	Yes	Yes	Yes	Yes	7
Liao (13)	Yes	Yes	Yes	Yes	Yes	Yes	No	Yes	No	7

**Table 3:** Summary of Findings Table.

Statin use and the active tuberculosis infection risk			
Patient or population: Overall population or patients with or without the use of statins Settings: Overall population (diabetic or non-diabetic, with or without specific disease), clinical settings Exposure: Statin use			
**Outcomes**	**Relative effect (95% CI)**	**No of Participants (studies)**	**Quality of the evidence (GRADE)**
Active tuberculosis infection ICD-9 or ICD-10 diagnosed Follow-up: 1~12 years	RR 0.60 (0.47 to 0.75)	2,133,735 (9 studies)	⊕⊝⊝⊝ low^a, b^
*The corresponding risk (and its 95% confidence interval) is based on the assumed risk in the comparison group and the relative effect of the intervention (and its 95% CI). CI: Confidence interval.			
GRADE Working Group grades of evidence High quality: Further research is very unlikely to change our confidence in the estimate of effect. Moderate quality: Further research is likely to have an important impact on our confidence in the estimate of effect and may change the estimate. Low quality: Further research is very likely to have an important impact on our confidence in the estimate of effect and is likely to change the estimate. Very low quality: We are very uncertain about the estimate.			

a *Inconsistency: A considerable heterogeneity was detected which could not be explained by gender difference, diabetic status, study design, or definition of study use*.

b *Indirectness: The validity of the definition of statin use and confirmation of active tuberculosis infection outcome were not consistently reported in registries*.

### Association Between Statin Use and Tuberculosis Infection

**Figure 2:**
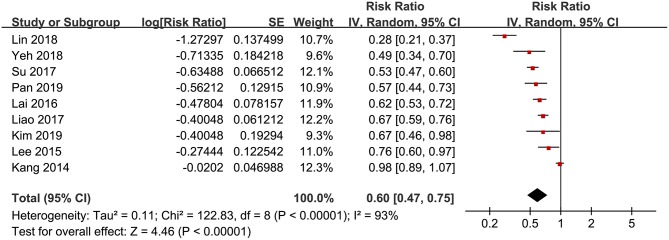
Forest plots for the meta-analysis of the association between statin use and active tuberculosis infection.

**Figure 3:**
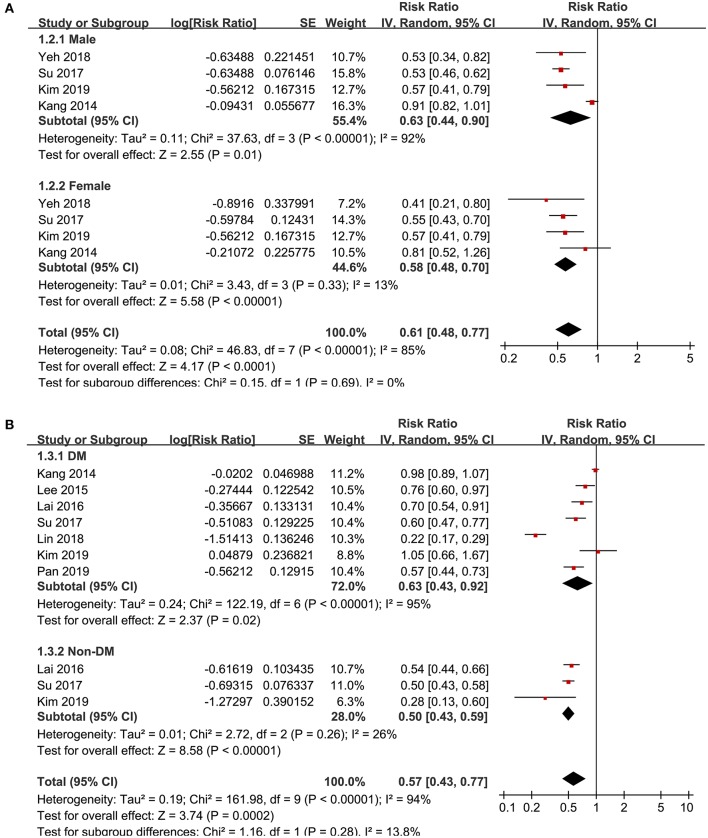
Subgroup analyses for the association between statin use and active tuberculosis infection. **(A)** Subgroup analyses according to the gender of the participants; and **(B)** subgroup analyses according to the diabetic status of the participants.

**Figure 4:**
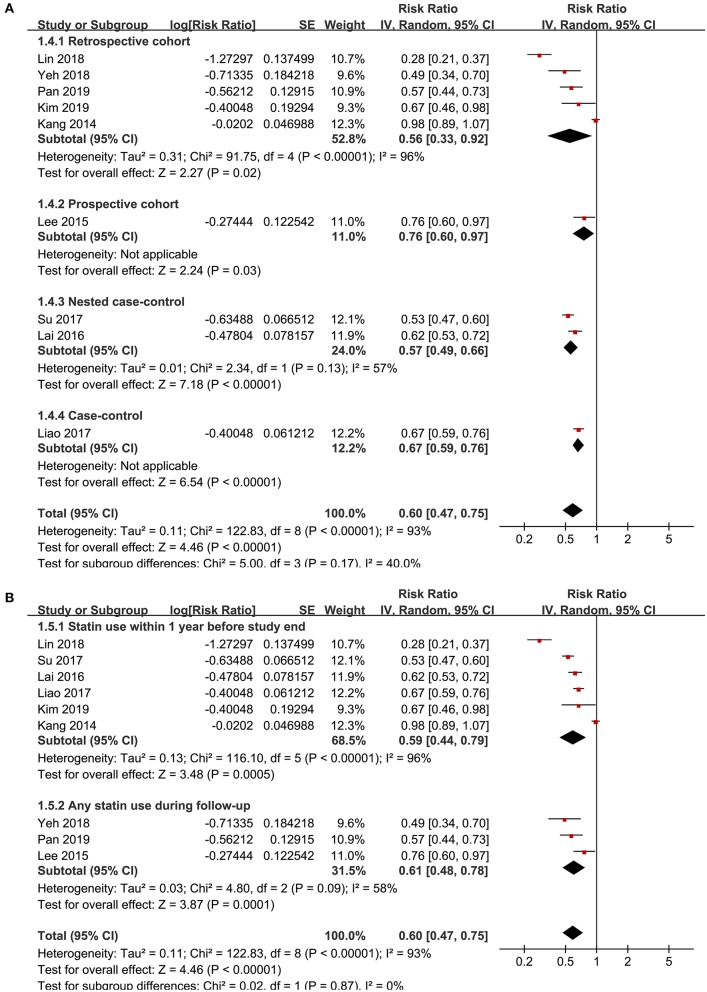
Subgroup analyses for the association between statin use and active tuberculosis infection. **(A)** Subgroup analyses according to the study design characteristics and **(B)** subgroup analyses according to the definition of statin use.

Significant heterogeneity was detected among the included studies (p for Cochrane's Q test <0.001, *I*^2^ = 93%), and a random-effect model was used to pool the results, which showed that that statin use was negatively associated with active tuberculosis infection (RR: 0.60, 95% CI: 0.45 to 0.75, *p* < 0.001; Figure 2). However, following the GRADE methodology, the quality of evidence was low (Table 3). Results of sensitivity analyses by omitting one study at a time did not significantly change the results (RR: 0.56~0.65, *p* all < 0.05). Particularly, meta-analysis limited to follow-up studies showed similar results [eight studies (10–12, 14–18), RR: 0.58, 95% CI: 0.44 to 0.77, *p* < 0.001]. Subgroup analyses showed that the negative association between statin use and active tuberculosis infection was consistent in men (RR: 0.63, 95% CI: 0.44 to 0.90, *p* = 0.01) and women (RR: 0.58, 95% CI: 0.48 to 0.70, *p* < 0.001; Figure 3A), and in participants with (RR: 0.63, 95% CI: 0.43 to 0.92, *p* = 0.02) and without diabetes (RR: 0.50, 95% CI: 0.43 to 0.59, *p* < 0.001; Figure 3B). Moreover, consistent results were obtained for retrospective cohort studies (RR: 0.56, *p* = 0.02; Figure 4A), prospective cohort studies (RR: 0.76, *p* = 0.03), nested case-controls studies (RR: 0.57, *p* < 0.001), and case-control studies (RR: 0.60, *p* < 0.001), and in studies with statin used defined as any use within 1 year (RR: 0.59, *p* < 0.001; Figure 4B) or during follow-up (RR: 0.61, *p* < 0.001).

### Publication Bias

**Figure 5:**
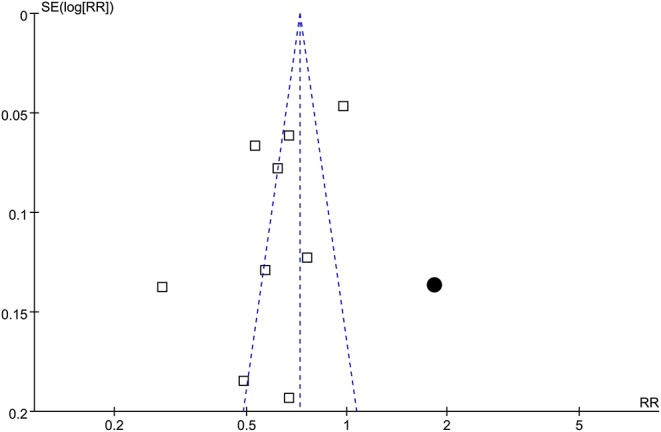
Funnel plots with trim-and-fill analyses for the publication bias underlying the meta-analysis of the association between statin use and active tuberculosis infection; the blank squares represent each of the included study, and the black circular indicates the imputed study with negative result.

The funnel plots regarding the association between statin use and active tuberculosis infection were shown in Figure 5. The funnel plots were asymmetry on visual inspection, suggesting high risk of publication bias. Results of Egger's regression test also suggested the possibility of significant publication bias (*p* = 0.046). Subsequently, we used the “trim and fill” analyses to incorporate an imputed study with negative finding to generate symmetrical funnel plots. Including this hypothesized study into the meta-analysis did not significantly change the result (RR: 0.72, 95% CI: 0.68 to 0.76, *p* < 0.001).

## Discussion

This meta-analysis showed that statin use may be associated with reduced active tuberculosis infection. The robustness of the finding was confirmed by the results of sensitivity analyses. Stratified analyses showed that the negative association between statin use and active tuberculosis infection was consistent regardless of the gender, diabetic status of the participants, study design, and definitions of statin use. However, significant heterogeneity was detected among the included studies, and the GRADE approach showed that the overall quality of the included studies for the meta-analysis is low. In addition, potential publication bias was detected, although subsequent meta-analysis by incorporating an imputed study retrieved by “trim-and-fill” analysis to generate symmetrical funnel plots also showed a negative association between statin use and active tuberculosis infection. Taken together, results of the meta-analysis demonstrated that statin use may be negatively associated with the risk of active tuberculosis infection. However, the low quality of the included studies, considerable heterogeneity, and potential publication bias lead to the uncertainty of the findings. Randomized controlled trials (RCTs) are needed to validate the potential preventative efficacy of statin therapy for active tuberculosis infection.

To the best of our knowledge, our study is the first meta-analysis to summarize the relationship between statin use and active tuberculosis infection based on epidemiological studies. The clinical implications mainly include the followings. Firstly, we found a possible negative association between statin use and active tuberculosis infection. The results were based on studies with adjustment of potential confounding factors including age, gender, comorbidities, and other concurrent medications. In addition, sensitivity analyses limited to follow-up studies showed consistent result. These results may suggest an independent association between stain use and reduced risk of active tuberculosis infection, highlighting the potential importance of statin use as a protective factor against tuberculosis infection. Finally, since gender difference for the prevalence of tuberculosis has been proposed (27), and diabetes has been recognized as a risk factor for tuberculosis infection (28), we analyzed whether the association between statin use and active tuberculosis infection varied according to the gender and diabetic status of the participants. Results showed that the association between statin use and reduced active tuberculosis infection was consistent regardless of the gender and diabetic status of the participants, which further confirmed the robustness of the results. In addition, subgroup analyses according to the characteristics of study design and definition of statin design were also performed, which showed that these factors did not affect the association between statin use and active tuberculosis infection. However, results of subgroup analyses did not support that any of the analyzed characteristics could contribute to the heterogeneity, including gender difference, diabetic status, study design, and definitions of statin use. Besides, in view of the significant heterogeneity among the included studies and potential risk of publication bias for the meta-analysis, current evidence supporting the negative association between statin use and active tuberculosis infection is limited in low-quality observational studies. In addition, difference among other study characteristics, such as the dose, treatment duration, and methods for the validation of active tuberculosis may contribute to the great heterogeneity among the included studies. However, since these characteristics were rarely reported in detail in the included studies, which prevented us from further analyses. Taken together, current evidence from limited low-quality observational studies indicated that statin use may be associated with reduced active tuberculosis infection. Future RCTs are needed to validate these findings.

The potential mechanisms underlying the negative association between statin and active tuberculosis infection may be multifactorial. Cholesterol is essential for the internalization of mycobacteria in host cells, including *M. tuberculosis* (29). Atorvastatin has been shown to inhibit cholesterol efflux in THP-1 macrophages (30), thereby potentially restraining the *M. tuberculosis* from internalization into the macrophages. Moreover, *in vitro* studies in peripheral blood mononuclear cells infected with *M. tuberculosis* showed that treatment with fluvastatin slightly induces the release of TH1 cytokines and promotes the activation of caspase 1, indicating that statins could strengthen the host response against *M. tuberculosis* (31). In addition, lovastatin and fluvastatin have both been reported to inhibit the activation of γδ T cells induced by *M. tuberculosis* antigens (32). Finally, vitamin D deficiency has been confirmed as an independent risk factor for tuberculosis infection in view of its role in regulation of immune response and anti-infection (33). Since treatments with atorvastatin and rosuvastatin have been shown to increase the serum vitamin D concentration (34), statins may be protective against tuberculosis infection via restoration of vitamin D level in the vulnerable population. Future studies are needed to clarify the key molecular mechanisms underlying the negative association between statin use and tuberculosis infection.

Our study has limitations which should be considered when interpreting the results. Firstly, significant heterogeneity exists among the included studies. Although subgroup analyses indicated that gender, diabetic status of the participants, study design, and definitions of stain use may not affect the results, other study characteristics may contribute to the heterogeneity, such as the dose and duration of statin use, concurrent using of some other medications which may affect the risk of tuberculosis infection [for example metformin (35), or proton pump inhibitor (36)] and the glycemic status of patients with diabetes (37). Moreover, since the individual patient data was not available, we could only perform subgroup analyses based on study-level data. The influences of patient and study characteristics on the negative association between statin use and active tuberculosis infection should be analyzed in future studies. Secondly, although we included studies with adjusted data for the association between stain use and active tuberculosis infection, we could not exclude the existence of residual factors which may confound the association. Thirdly, all of the included studies enrolled Asian participants. The association between statin use and active tuberculosis infection in participants of other ethnicities should be also evaluated. Fourthly, a causative association between statin use and decreased active tuberculosis infection should not be derived based on our finding since this study was a meta-analysis of observational studies. Moreover, we could not determine whether the dosages, durations, or using individual statin medications may affect the negative association between statin use and active tuberculosis infection. Finally, as previously mentioned, this meta-analysis was based on low-quality observational studies, with considerable heterogeneity, and possible publication bias, which highlights the necessity of future RCTs to validate the finding.

In conclusion, statin use may be negatively associated with active tuberculosis infection. However, substantial heterogeneity was detected and the level of the evidence for such preventative effect from included studies was low. Future RCTs are needed to confirm the potential preventative role of statin use on tuberculosis infection.

## Data Availability Statement

All datasets generated for this study are included in the article/supplementary material.

## Author Contributions

XL and LL conceived and designed the study and analyzed data, and all authors interpreted the results. XL and LS selected the studies and collected the data and drafted and revised the paper. All authors revised the draft paper. All authors read and approved the final version of the manuscript.

## Conflict of Interest

The authors declare that the research was conducted in the absence of any commercial or financial relationships that could be construed as a potential conflict of interest.
